# Simplified technique for DaVinci view spine presentation

**DOI:** 10.1186/1748-7161-9-S1-O20

**Published:** 2014-12-04

**Authors:** Paweł Główka, Tomasz Kotwicki

**Affiliations:** 1 Department of Pediatric Orthopaedics and Traumatology, University of Medical Sciences, Poznan, Poland

## Background

DaVinci presentation is a physician friendly picture of scoliotic spine seen from the cephalad side in the horizontal plane. This presentation is complementary to the standard view of the spine in two planes: frontal (anterior-posterior) and sagittal (lateral). DaVinci presentation can be obtained by using a few techniques which allow for three-dimensional reconstructions: CT, MRI, EOS. DaVinci-presentation is useful in analyzing the spinal deformation in 3D space, in horizontal plane: the deviation of each vertebrae in relation to the saggital plane, CLS (center sacral line) or C7PL (C7 plumbline); localization of PMC (plane of maximum curvature) in relation to the saggital and coronal plane.

## Aim

Create the simple way to draw the DaVinci-presentation on the basis of X-rays (AP, lat) without the necessity of using the sophisticated software.

## Design

Draw the DaVinci-presentation of scoliosis manually. Validate the drawing method.

## Material

X-rays and CT scans of three patients with scoliosis. The magnitude of the main curve, assessed by the Cobb's angle, amounted to: 71, 87, 88 degrees respectively, 82 on average.

## Methods

To create the DaVinci-presentation the central points of vertebrae were determined. The method used to define the vertebrae's central points and to draw the DaVinci-presentation was validated. In the first validation's step the virtual model of spine was created. This model was subjected to mathematical analysis. In the second validation's step, the accuracy of assessed position of 42 vertebrae's centers was verified by examining the CT-scans of analyzed curves.

## Results

DaVinci presentations of analysed scolioses were drawn. The localisation of central points of vertebrae were close to their real position.

## Conclusion

Simplified way to draw a DaVinci presentation on the basis of regular AP and lateral X-rays is possible.
